# Osteogenesis from Dental Pulp Derived Stem Cells: A Novel Conditioned Medium Including Melatonin within a Mixture of Hyaluronic, Butyric, and Retinoic Acids

**DOI:** 10.1155/2016/2056416

**Published:** 2016-01-10

**Authors:** Margherita Maioli, Valentina Basoli, Sara Santaniello, Sara Cruciani, Alessandro Palmerio Delitala, Roberto Pinna, Egle Milia, Regina Grillari-Voglauer, Vania Fontani, Salvatore Rinaldi, Roberta Muggironi, Gianfranco Pigliaru, Carlo Ventura

**Affiliations:** ^1^Department of Biomedical Sciences, University of Sassari, Viale San Pietro 43/B, 07100 Sassari, Italy; ^2^Laboratory of Molecular Biology and Stem Cell Engineering, National Institute of Biostructures and Biosystems, Via Massarenti, 40138 Bologna, Italy; ^3^Research Department, Rinaldi Fontani Foundation, Viale Belfiore 43, 50144 Florence, Italy; ^4^Department of Biotechnology, University of Natural Resources and Life Sciences Vienna, Muthgasse 18, A-1190 Vienna, Austria; ^5^Azienda Ospedaliera Universitaria di Sassari, Via Michele Coppino 26, 07100 Sassari, Italy; ^6^Department of Regenerative Medicine, Rinaldi Fontani Institute, Viale Belfiore 43, 50144 Florence, Italy; ^7^Stem Wave Institute for Tissue Healing (SWITH), Gruppo Villa Maria and Ettore Sansavini Health Science Foundation, Via Provinciale per Cotignola 9, 48022 Lugo, Ravenna, Italy

## Abstract

Human dental pulp stem cells (hDPSCs) have shown relevant potential for cell therapy in the orthopedic and odontoiatric fields. The optimization of their osteogenic potential is currently a major challenge. Vascular endothelial growth factor A (VEGF A) has been recently reported to act as a major conductor of osteogenesis* in vitro *and* in vivo*. Here, we attempted to prime endogenous VEGF A expression without the need for viral vector mediated gene transfer technologies. We show that hDPSCs exposure to a mixture of hyaluronic, butyric, and retinoic acids (HA + BU + RA) induced the transcription of a gene program of osteogenesis and the acquirement of an osteogenic lineage. Such response was also elicited by cell exposure to melatonin, a pleiotropic agent that recently emerged as a remarkable osteogenic inducer. Interestingly, the commitment to the osteogenic fate was synergistically enhanced by the combinatorial exposure to a conditioned medium containing both melatonin and HA + BU + RA. These* in vitro* results suggest that* in vivo* osteogenesis might be improved and further studies are needed.

## 1. Introduction

Mesenchymal stem cells (MSCs) are distributed in different tissues, including bone marrow, brain, skin, adipose tissue, and have the capability to differentiate towards specific phenotypes to replace damaged cells. Within this context, human dental pulp MSCs (hDPMSCs) can easily be isolated [1, 2] and represent a valuable source of readily accessible stem cells for future strategies of tissue engineering or cell therapy. The ability of hDPSCs to differentiate into neurogenic, osteogenic/odontogenic, adipogenic, myogenic, and chondrogenic lineages has been confirmed in recent studies [2, 3]. In addition to their ability to move towards so many lineages, hDPSCs have been regarded as an attractive tool to accomplish stem cell commitment into odontoblasts capable of depositing mineralized matrix [4] and to pursue studies on the biological compatibility of innovative dental materials [5]. MSC differentiation is finely regulated by the action of physical and chemical signals [6–8] which can be artificially induced. Dexamethasone, ascorbic acid, and beta-glycerophosphate have all been extensively investigated for their ability to induce osteogenesis. Compounding the list of agents that may play a crucial role in this process, melatonin, an indolamine influencing circadian rhythms, sleep-wake cycle, tumor growth inhibition, and immune function [9–11], has been recently described as a regulator of stem cell differentiation, being an enhancer of osteogenesis from bone marrow stem cells, while inhibiting adipogenesis [12, 13]. Several studies also demonstrated that melatonin prevents bone deterioration including idiopathic scoliosis in adolescents and that it stimulates osteoblastic differentiation [14, 15].

However, the development of novel conditioned media affording a high-throughput of osteogenesis in hDPSCs remains highly demanding and represents a remarkable challenge.

To this end, vascular endothelial growth factor A (VEGF A) has been recently included among the list of growth factors behaving as specific osteogenic inducers, such as FGF-2 and Bmp-2, being able to act as an osteogenesis orchestrator during fracture healing* in vivo* [6, 16, 17].

We have previously synthesized a mixed ester of hyaluronic acid (HA) with butyric acid (BA) and retinoic acid (RA) [7, 18], providing evidence that this compound, not only primed cardiogenesis in mouse embryonic stem cells [7, 18], also afforded a remarkable increase in VEGF A gene and protein expression in hMSCs isolated from the bone marrow and alternative sources, including the dental pulp, and term placenta [6], enhancing hMSC-mediated cardiovascular repair* in vivo* in a rat model of myocardial infarction [7]. More recently, we observed that a consistent increase in VEGF A gene transcription and angiogenic commitment can also be induced by a mixture of HA, BU, and RA (HA + BU + RA) in both human adipose-derived stem cells [8] and amniotic fluid stem cells [8].

Here, we aimed at investigating whether exposure in the presence of the HA + BU + RA mixture or melatonin may efficiently promote an osteogenic patterning in hDPSCs and whether, in the affirmative, the commitment to the osteogenic fate may be turned into a high-throughput process by cell exposure to a combination of both conditioned media.

## 2. Materials and Methods

### 2.1. Cell Isolation

Vital human molars were obtained from 10 adult subjects (age 15–26) during a routine dental extraction, and the dental pulp was used to isolate stem cells according to the approval of the local ethical committee (ref. number 2034/2014). The teeth were immersed in physiological solution containing antibiotics. After extraction, the dental crown was fractured in several parts by means of pliers (bone forceps) under sterile conditions, and the pulp was uncovered. The tissue fragments were suspended in DMEM in the presence of 25% FBS, antibiotics (200 units/mL penicillin, 100 *μ*g/mL streptomycin), and 2.5 *μ*g/mL amphotericin B. This procedure was followed by incubation at 37°C in a humidified atmosphere containing 5% CO2, allowing cells to slip down from the explants and expand up to confluence. The cell layer was removed by enzymatic digestion (0.08% trypsin, EDTA) at 37°C and further expanded in medium containing DMEM, 15% fetal bovine serum (FBS) and 2 mM glutamine, 100 U/mL penicillin and 100 *μ*g/mL streptomycin, 1 mM Nonessential Amino Acids, 1 mM sodium pyruvate, and 1 mM beta-mercaptoethanol (culturing medium C). The hDPSCs were immunomagnetically sorted for c/kit using a monoclonal anti-c/kit (CD117) antibody (Miltenyi Biotech, Minneapolis, MN, USA) directly conjugated to MicroBeads (Miltenyi Biotec) and then expanded in subconfluent conditions in medium C. All cells used for experimentation were not frozen and were treated between passages 4 and 8.

### 2.2. Cell Characterization and Culturing

The fibroblast-like cells obtained from dental pulp, cultured at the same doubling passage, were harvested by treatment with 0.08% trypsin and EDTA and incubated with 1 *μ*g/10^6^ cells of fluorescein isothiocyanate-conjugated antibodies for 40 min at 4°C in the dark. The antibodies used were SH2, SH3, SH4, anti-CD166, anti-CD14, anti-CD34, anti-CD44, and anti-CD45. After washing, cells were analyzed on a flow cytometer (FACSCalibur, BD Biosciences) by collecting 10,000 events, and the data were analyzed using the CellQuest Software (BD Biosciences). All cells used in this study were positively stained for CD90, CD105, CD44, and CD29, lacking the expression of CD34, CD133, and CD45 (data not shown). To obtain osteogenic differentiation, cells from eight donors were separately seeded at 8,000 cells/cm^2^ in 4 different conditioned media: (i) medium C + 0,01 M melatonin (medium M); (ii) medium C + a mixture of HA (2 mg/mL) + BU (5 mM) + RA (1 *μ*M), all from Sigma-Aldrich (medium H); (iii) medium C + a mixture of HA (2 mg/mL) + BU (5 mM) + RA (1 *μ*M) + 0,01 M melatonin (medium MH); and (iv) dexamethasone-based medium (medium D), containing D-MEM low glucose, 10% fetal bovine serum, 100 nM dexamethasone, 200 *μ*M L-Ascorbic acid 2-phosphate, 10 mM beta-glycerol 2-phosphate, 2 mM glutamine, 100 U/mL penicillin, and 100 *μ*g/mL streptomycin. Control cells from two donors were cultured only in medium C and kept at undifferentiated state. HA, BU, and RA and melatonin were dissolved in water and were added every 24 hours.

### 2.3. Mineralization

To assess the mineralization, hDPMSCs were cultured for 7, 14, or 21 days in the absence (control) or presence of one of the above mentioned conditioned media (medium M, medium H, or medium MH). Control undifferentiated cells were cultured in cells that were maintained in medium C. Mineralization/calcium deposition was quantified by Alizarin red stain. The samples were washed three times in distilled water (ddH2O), and then cells were stained with 2% alizarin red S solution (Santa Cruz Biotechnology) for 30 min at room temperature. Samples were then thoroughly washed five times in ddH2O to avoid excess solution leftover on samples and incubated in PBS for 15 min before being air-dried in a fume hood. Cells were observed by the optical microscope Leica DM IL. The nodules were analyzed using image analysis software (ImageJ, National Institutes of Health) to determine the number and area of mineralized nodules.

### 2.4. Gene Expression Analysis

Total RNA was isolated from adherent cells (Monolayer) cultured in medium C and kept at undifferentiated state or from cells exposed for 1, 3, 7, 14, or 21 days in presence of one of the 4 conditioned media (medium D, medium M, medium H, or medium MH), by the standard TRIzol Reagent extraction, according to the manufacturer's instructions (cat#15596-026, Life Technologies). RNA was dissolved in diethylpyrocarbonate- (DEPC-) treated water. Total RNA was treated with 1 U of Turbo RNase-free DNase (TURBO DNA-free Kit, cat#AM1907, Life Technologies) and reverse transcribed into cDNA using SuperScript VILO cDNA Synthesis Kit (cat#11754, Life Technologies). An amount of total RNA (2.5 *μ*g) was added to a reaction mixture, 5x VILO Reaction Mix, including random primers, MgCl_2_, and dNTPs, 10x SuperScript Enzyme Mix including SuperScript III RT, and RNaseOUT. The RT reaction was carried out at 25°C for 10 min, followed by 1 h at 42°C, and the reaction was stopped by heating for 5 min at 85°C. Afterwards, the reaction tubes containing RT preparations were flash-cooled in an ice chamber until being used for DNA amplification through semiquantitative real time-polymerase chain reaction (sqRT-PCR). sqRT-PCR reactions were set up in 25 *μ*L reaction mixtures containing Platinum Quantitative PCR SuperMix-UDG (cat#11743-100, Life Technologies), SYBR, fluorescein NIST-traceable standard primer, distilled water, and cDNA template. The reaction program was allocated to the first step and it was at 50°C for 2 minutes' hold (UDG incubation), 95°C for 2 minutes' hold and 40 cycles of 95°C for 15 seconds, 50–56.0°C for 30 s, and 60°C for 30 seconds. At the end of each sqRT-PCR, a melting curve analysis was performed at 95.0°C to check the quality of used primers. Each experiment included a distilled water control. The sqRT-PCR analysis was performed for the following set of genes: VEGF A, Runt-related transcription factor 2 (Runx2), Zinc finger and BTB domain containing protein 16 (ZBTB16), nuclear receptor subfamily 4, group A, member 3 (NR4A3), stanniocalcin 1 (STC1), osteocalcin (OCN), bone sialoprotein (BSP), alkaline phosphatase (ALP), Nanog Homeobox (Nanog), SRY (sex determining region Y) box 2 (Sox2), and octamer-binding transcription factor 4 (Oct4). The relative expression of each transcript was determined using the delta-CT method, with glyceraldehyde 3-phosphate dehydrogenase (GAPDH) as a reference gene. All primers used were from Life Technologies and are reported in Table 1.

### 2.5. Immunostaining

Cells were cultured for a period of 7, 14, or 21 days in 3 different conditioned media: (i) medium C + melatonin (medium M), (ii) medium C + HA + BU + RA mixture (medium H), and (iii) medium C + melatonin + HA + BU + RA mixture (medium MH). Control cells were maintained undifferentiated in the presence of medium C. After 7, 14, or 21 days, the cells were treated with trypsin, and the resulting suspension was cultured at low density to allow visualization of individual cells. Cells were fixed with 100% methanol at −20°C for 30 min and then at −80° for 30 min. After permeabilization by PBS containing 0.25% Triton X-100, cells were washed in PBS three times for 5 min and incubated with 1% BSA in PBS-T (PBS + 0.1% Tween 20) for 30 min and then exposed overnight at 4°C to the primary anti-mouse monoclonal antibodies BSPII (Santa Cruz Biotechnology), osteocalcin (Santa Cruz Biotechnology), and Runx2 (Santa Cruz Biotechnology) or to the rabbit polyclonal antibodies against VEGF A (Thermo Scientific). Cells were then washed in PBS three times for 5 min and stained at 37°C for one hour in the dark with the fluorescence-conjugated goat IgG secondary antibody. All microscopy analyses were performed with a confocal microscope (TCS SP5, Leica, Nussloch, Germany). DNA was visualized with 1 *μ*g/mL 4,6-diamidino-2-phenylindole (DAPI).

### 2.6. Statistical Analysis

Data were analyzed using IBM SPSS software, version 22. The congruity of the data was evaluated with the Wilcoxon test. The congruity of the treatments was further assessed with Kruskal-Wallis test and then Jonckheere-Terpstra test, assuming a *P* value less than 0.005 to be the limit of significance.

## 3. Results

### 3.1. A Mixture of Melatonin with HA, BU, and RA Enhances the Expression of Osteogenic Genes

**Figure 1:**
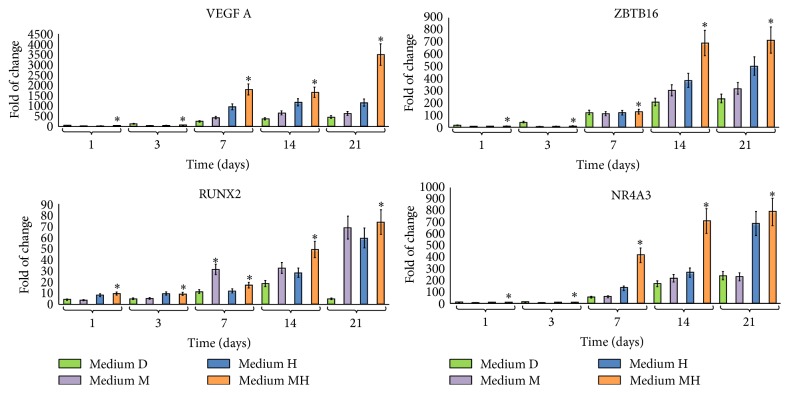
Effect of melatonin and HA + BU + RA exposure on the expression of genes orchestrating hDPSCs commitment toward an osteogenic lineage. Cells were exposed for 1, 3, 7, 14, or 21 days in the presence of one of the 4 conditioned media described under Materials and Methods and based on 100 nM dexamethasone with 200 *μ*M L-Ascorbic acid 2-phosphate (medium D), 0,01 M melatonin (medium M), or a mixture of HA 2 mg/mL + BU 5 mM + RA 1 *μ*M (medium H) or on a combination of melatonin together with HA + BU + RA (medium MH) or kept at the undifferentiated state by growing on medium C. The amounts of vascular endothelial growth factor A (VEGF A), Runx2, ZBTB16, and NR4A3 mRNA from treated or untreated cells were normalized to GAPDH, and the mRNA expression of cells differentiated with the 4 different conditioned media (D, M, H, and MH) was plotted at each time point as fold change relative to the expression in control undifferentiated cells defined as 1 (mean ± SE; *n* = 6). All data from medium D-treated cells, M-treated cells, medium H-treated cells, and medium MH-treated cells at each time point were significantly different from those in control undifferentiated cells. mRNA levels from cells that had been exposed to medium D, medium M, medium H, and medium MH were significantly different from each other at each time point. ^*∗*^Significantly different from medium D-treated cells. Medium D: green bars, medium M: purple bars, medium H: blue bars, and medium MH: orange bars.

**Figure 2:**
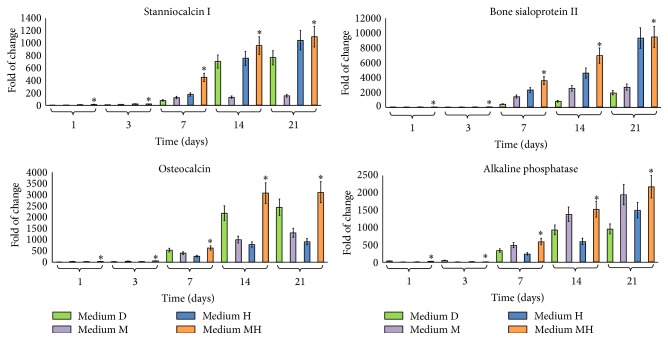
Effect of melatonin and HA + BU + RA on the expression of osteogenic specific genes. Cells were exposed for 1, 3, 7, 14, or 21 days in the presence of one of the 4 conditioned media described under Materials and Methods and based on 100 nM dexamethasone with 200 *μ*M L-Ascorbic acid 2-phosphate (medium D), 0,01 M melatonin (medium M), or a mixture of HA 2 mg/mL + BU 5 mM + RA 1 *μ*M (medium H) or on a combination of melatonin together with HA + BU + RA (medium MH) or kept at the undifferentiated state by growing on medium C. The amounts of stanniocalcin 1 (STC1), osteocalcin, bone sialoprotein II (BSPII), and alkaline phosphatase mRNA from treated or untreated cells were normalized to GAPDH, and the mRNA expression of cells differentiated with the 4 different conditioned media (D, M, H, and MH) was plotted at each time point as fold change relative to the expression in control undifferentiated cells, defined as 1 (mean ± SE; *n* = 6). All data from medium D-treated cells, M-treated cells, medium H-treated cells, and medium MH-treated cells at each time point were significantly different from those in control undifferentiated cells. mRNA levels from cells that had been exposed to medium D, medium M, medium H, and medium MH were significantly different from each other at each time point. ^*∗*^Significantly different from medium D-treated cells. Medium D: green bars, medium M: purple bars, medium H: blue bars, and medium MH: orange bars.

The exposure of hDPSCs to each of the conditioned media D, M, or H, containing dexamethasone, melatonin, or a mixture of HA + BU + RA, respectively, resulted in a significant increase in the transcription of VEGF A (Figure 1). Interestingly, hDPSCs, treated with medium MH, exhibited higher VEGF A mRNA levels than cells cultured in the presence of medium M or medium H alone or in medium D indicating that melatonin and HA + BU + RA can exert synergistic effects on VEGF A transcription (Figure 1). The same figure also shows the effects elicited by melatonin or a mixture of HA + BU + RA on the gene of expression of the Runx2, ZBTB16, and NR4A3, acting as key regulators of chondroblast and osteoblast differentiation, controlling osteogenic specific genes such as alkaline phosphatase, and extracellular matrix mineralization [19]. Noteworthy, cell exposure to the combination of HA + BU + RA mixture with melatonin (medium MH) significantly increased (mean ± SE; *n* = 6; *P* < 0.05) the expression of Runx2, ZBTB16, and NR4A3, as compared to cells treated with either of the conditioned mediums alone (medium M or medium H), including the conventional dexamethasone-based one (Figure 1). The combination of the two conditioned media (medium MH) was also found to remarkably induce the transcription of the lineage-specific genes stanniocalcin 1 (STC1), osteocalcin (OCN), bone sialoprotein II (BSPII), and alkaline phosphatase, with the effect being particularly evident after 14 days of treatment (Figure 2). Interestingly, medium MH elicited the most remarkable increase in the gene expression of all the investigated late markers of osteogenic specification (Figure 2).

### 3.2. Exposure to a Mixture of Melatonin with HA, BU, and RA Modulates the Expression of Osteogenic Specific Marker Proteins

**Figure 3:**
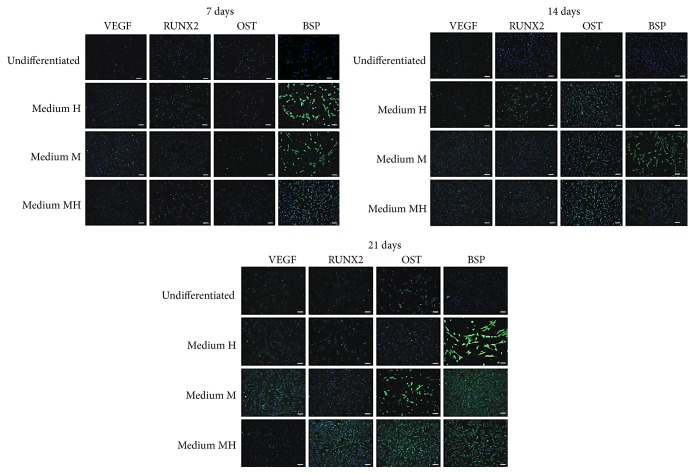
Analysis of osteogenic specific proteins. Expression of VEGF A, Runx2, osteocalcin (OCN), and bone sialoprotein (BSP) was assessed in cells cultured for 7, 14, or 21 days in the presence of one of the 3 conditioned media described under Materials and Methods and based on 0,01 M melatonin (medium M) or a mixture of HA 2 mg/mL + BU 5 mM + RA 1 *μ*M (medium H) or on a combination of melatonin together with HA + BU + RA (medium MH) or kept under an undifferentiated state by growing on medium C. Nuclei are labeled with 4,6-diamidino-2-phenylindole (DAPI, blue). Scale bars: 40 *μ*m. The figures are representative of five separate experiments. For each differentiation marker, fields with the highest yield of positively stained cells are shown.

Immunofluorescence assays revealed that hDPMSC exposure to melatonin (medium M) or HA + BU + RA (medium H) consistently increased the number of cells expressing VEGF A, RUNX2, osteocalcin, and bone sialoprotein, as compared with control untreated cells (Figure 3). Such an increase was already evident after 7 days of treatment and persisted during the following 14 days in culture for all the investigated proteins (Figure 3). Consonant with the gene expression observations, the conditioned medium containing HA + BU + RA plus melatonin (medium MH) was able to induce a higher yield of cells exhibiting an osteogenic phenotype, compared with DPSCs cultured with each conditioned medium alone (Figure 3).

### 3.3. DPSCs Treated with Melatonin in Combination with HA, BU, and RA Exhibit High Differentiation Rate toward the Osteogenic Phenotypes

**Figure 4:**
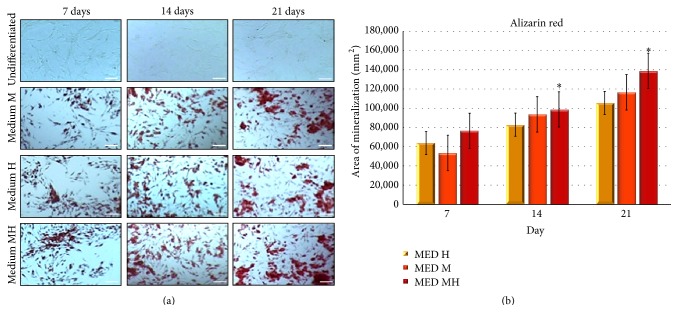
Alizarin red stain quantified by measurement of calcium deposits. (a) hDPSCs were cultured for 7, 14, or 21 days in the presence of one of the 3 conditioned media described under Materials and Methods and based on 0,01 M melatonin (medium M) or a mixture of HA 2 mg/mL + BU 5 mM + RA 1 *μ*M (medium H) or on a combination of melatonin together with HA + BU + RA (medium MH) or kept under an undifferentiated state by growing on medium C. Scale bar = 200 um. (b) Quantitative mineralization of hDPSCs cultured in maintenance medium (medium C) or the different osteogenic media (medium M, medium H, and medium MH). Results are expressed as mean + SD. An average was made from three technical replicates. Values represent the mean standard deviation of 4 different experiments.

To further evaluate the effect of the currently developed conditioned media on osteogenic differentiation, we assessed the number of alizarin red positive cells throughout a period of 21 days of culture in the absence or presence of media M, H, or MH.  Figure 4 shows that after 21 days of exposure the combined mixture in medium MH acted as a more effective osteogenic inducer, compared with medium M or medium H alone.

## 4. Discussion

Modulation of the osteogenic potential in human mesenchymal stem cells has become a relevant topic in orthopedics and in the odontoiatric field. Human dental pulp is now regarded as an interesting reservoir of stem cells highly amenable for regenerative medicine for both oral and nonoral diseases, owing to a number of well-established features of DPSCs, including multipotency, high proliferation, and accessibility. Although future clinical applications of these cells would also rely on their embedding within defined transplantable scaffolds with tailored oriented architecture, the optimization of the osteogenic potential in DPSCs will form an essential cue for the development of successful tissue engineering/regenerative strategies.

In the attempt to afford a high-throughput of osteogenesis in hDPSCs, we investigated their response to melatonin, a mixture of HA + BU + RA, or a combination of the two conditioned media. Mesenchymal stem cells are currently committed toward the osteogenic lineage mainly by a dexamethasone-conditioned medium, even though also melatonin has been shown to induce the Runx2-dependent osteogenic pathway [12]. We have previously shown that HA, BU, and RA are able to induce the expression of cardiovascular orchestrating genes, including VEGF A, and that the effect is maximum when the three compounds are combined together [8]. Recent evidence indicates that VEGF A is an important regulator of osteogenesis, by mediating bone vascularization and differentiation towards osteoblasts [16]. Here, we detected VEGF A gene expression in cells committed to osteogenesis, by the different conditioned media based upon melatonin (medium M), on a mixture of HA + BU + RA (medium H), or on a combination of all of these compounds (medium MH). Although all conditioned media were able to induce VEGF A gene expression, the effect was of significantly greater magnitude when hDPSCs were treated in the presence of the MH combination of conditioned media. Our finding that such synergistic effect occurred at the transcriptional level and involved the intracellular overexpression of VEGF A itself is worthy of consideration, based upon the recent finding that VEGF A also acts intracellularly and not only as a secreted protein to regulate the balance of osteogenic patterning [17, 20]. In fact, this growth factor has been shown to mediate the effect of lamin A on mesenchymal stem cell differentiation, as revealed by the finding that cells from heterozygous lamin A-deficient mice express reduced levels of both VEGF A and the osteoblast transcription factor RUNX2 [17, 20]. This is associated with impaired differentiation into osteoblasts. Knock-down of VEGF A in bone marrow MSCs produced the same downregulatory action on RUNX2 gene expression as reducing lamin A levels [17, 20]. Further studies are in progress to assess whether the currently observed effect of MH medium on VEGF A transcription may involve a fine balance of osteogenesis and adipogenesis through lamina A and/or other proteins forming the linker between cytoskeleton and nucleoskeleton.

The transcriptional enhancement in both ZBTB16 and NR4A3 in medium MH-treated cells observed in comparison to cells exposed to each medium alone is also relevant, due (i) to the essential role of these players in the orchestration of stem cell osteogenesis and (ii) to the ability of ZBTB16, a zinc finger and BTB domain containing protein 16, to fashion an osteogenic program independently of Runx2, as it has been shown in dexamethasone-induced osteogenesis [21]. Within this context, the enhancement in osteogenic commitment observed in the presence of MH medium may also be partially explained by the observation that while melatonin mainly induced the expression of Runx2, HA + BU + RA resulted in being more efficient in inducing ZBTB16 and NR4A3 transcription. Both melatonin and HA + BU + RA-conditioned media elicited the expression of the specific osteogenic marker genes osteocalcin, stanniocalcin 1, bone sialoprotein, and alkaline phosphatase. The fact that the inductive effect of MH was particularly evident in osteocalcin gene expression may add further insights into the mechanism(s) underlying the osteogenic action of this conditioned medium. In fact, osteocalcin gene expression and secretion have been found to represent an early marker in mesenchymal stem cell osteogenesis* in vitro*, preceding biomineralization and also representing a marker for predicting* in vivo* osteogenic potential in bone tissue engineering [22].

On the whole, the ability of MH conditioned medium to optimize the osteogenic potential of hDPSCs was confirmed by confocal microscopy analysis of osteogenic and bone-restricted marker proteins, showing that the gene expression program modulated by this medium was translated into a phenotypic osteogenic commitment at the intact cell level.

## 5. Conclusion

Scientific research clearly indicates that the use of MSCs for the reconstruction and repair of bone is actually feasible. Some problems however deal with the commitment to the differentiated state, the persistence of the transplanted cells* in vivo*, vascularization, and integration with the recipient bone. In the present study, we describe a novel conditioned medium based upon the presence of both melatonin and a mixture of HA + BU + RA, which can induce osteogenesis with high efficiency. In this regard, it has been previously shown that mesenchymal stem cells exposed to a mixture of HA + BU + RA cotransplanted with islets improved islet graft revascularization and function in diabetic rats [23]. Moreover, these molecules have coaxed both murine embryonic and human mesenchymal stem cells towards the cardiovascular phenotype, remarkably enhancing the rescuing potential of human stem cells in* in vivo* animal models of myocardial infarction [7]. Similarly other authors found that melatonin pretreatment greatly increased survival of mesenchymal stem cells* in vitro *and reduced their apoptosis after transplantation into ischemic brain, by inducing VEGF A gene expression and angiogenesis [24]. Our* in vitro* results suggest that the combinatorial use of HA + BU + RA with the natural hormone melatonin might improve* in vivo* osteogenesis. Further studies are needed.

## Figures and Tables

**Table 1:** Primers used.

Primer name	Forward	Reverse
hGAPDH	GAGTCAACGGATTTGGTCGT	GACAAGCTTCCCGTTCTCAG
OCT4	GAGGAGTCCCAGGACATCAA	CATCGGCCTGTGTATATCCC
SOX2	CCGTTCATGTAGGTCTGCGAGCTG	CAACGGCAGCTACAGCATGATGC
NANOG	CATGAGTGTGGATCCAGCT	CCTGAATAAGCAGATCCAT
BONE SIALOPROTEIN 2	GAAGAAGAGGAGACTTCAAATG	TATCCCCAGCCTTCTTGGGA
ALKALINE PHOSPHATASE	CAACCCTGGGGAGGAGAC	GCATTGGTGTTGTACGTCTTG
OSTEOCALCIN	GAGCCCCAGTCCCCTACCCG	GACACCCTAGACCGGGCCGT
STANNIOCALCIN 1	TTCGGAGGTGCTCCACTTTC	CAGGCTTCGGACAAGTCTGT
ZBTB16	CAAGAAGTTCAGCCTCAAGCA	CACTCAAAGGGCTTCTCACC
NR4A3	CTCATTGGTGCGTCTCCTGT	CGTAGTTGCTCGAGYAGCCC
RUNX2	CTGTGCTCGGTGCTGCCCTC	TCGTCCACTCCGGCCCACAA
VEGFA	GCCAAGTGGTCCCAGGCTGC	TCGTCATTGCAGCAGCCCCC
